# CD73-A2a adenosine receptor axis promotes innate B cell antibody responses to pneumococcal polysaccharide vaccination

**DOI:** 10.1371/journal.pone.0191973

**Published:** 2018-01-29

**Authors:** David Allard, Roxanne Charlebois, Loise Gilbert, John Stagg, Pavel Chrobak

**Affiliations:** Centre de Recherche du Centre Hospitalier l’Université de Montréal (CRCHUM), Faculté de Pharmacie de l’Université de Montréal et Institut du Cancer de Montréal, Montréal, Quebec, Canada; Instituto Butantan, BRAZIL

## Abstract

Many individuals at risk of streptococcal infection respond poorly to the pneumococcal polysaccharide vaccine Pneumovax 23. Identification of actionable pathways able to enhance Pneumovax responsiveness is highly relevant. We investigated the contribution of the extracellular adenosine pathway regulated by the ecto-nucleotidase CD73 in Pneumovax-induced antibody responses. Using gene-targeted mice, we demonstrated that CD73-or A2a adenosine receptor deficiency significantly delayed isotype switching. Nevertheless, CD73- or A2aR- deficient adult mice ultimately produced antigen-specific IgG3 and controlled *Streptococcus pneumoniae* infection as efficiently as wild type (WT) mice. Compared to adults, young WT mice failed to control *S*. *pneumoniae* infection after vaccination and this was associated with lower levels of CD73 on innate B cells. We hypothesized that pharmacological activation of A2a receptor may improve Pneumovax 23 immunization in young WT mice. Remarkably, administration of the A2a adenosine receptor agonist CGS 21680 significantly increased IgG3 responses and significantly enhanced survival after *S*. *pneumoniae* challenge. Our study thus suggests that pharmacological activation of the A2a adenosine receptor could improve the efficacy of Pneumovax 23 vaccination in individuals at risk of streptococcal infection.

## Introduction

Infections with *S*. *pneumoniae* are a major cause of morbidity and mortality in infants under 2 years of age, elderly patients and immunocompromised individuals [1]. Studies in mice demonstrated that antibodies produced by B-1a, B-1b and marginal zone (MZ) innate B cells play an important role in T cell-independent (TI) immune control of this pathogen both in naïve mice and in mice vaccinated with pneumococcal polysaccharides [2, 3]. B-1a B cells contribute mostly by producing natural IgM Ab, while B-1b B cells and MZ B cells in addition to producing IgM can also isotype switch and produce IgG (mainly IgG3). While the role of human counterparts of B-1 B cells in anti-pneumococcal immunity still remains controversial [4, 5], several studies concluded that human B-1 B cells indeed constitute a major B cell population responding to Pneumovax 23 vaccination [4, 6].

While pneumococcal polysaccharide vaccination is effective in preventing *S*. *pneumoniae* infections, individuals who are at the highest risk of infection respond poorly to the Pneumovax 23 vaccine. For instance, elderly patients have a decreased B-1 B cell pool [7] and young infants are incapable of generating protective antibodies, suggesting impairment of Pneumovax specific B cells in these populations [8]. These observations stress the importance of identifying pathways and molecular targets which could be modulated therapeutically in order to enhance immune responses of these cells.

One of the important immune regulatory mechanism is through the production of extracellular adenosine by ecto-nucleotidases [9, 10]. Extracellular adenosine acts as a negative regulator of innate and adaptive immune responses and of inflammation. It exerts many of its immunoregulatory effects through the A2a receptor (one of the four adenosine receptors) and modulates multiple aspects of immune responses, including immune cell effector and regulatory functions, and cell homing [11, 12]. Therapeutic modulation of the adenosine pathway is an increasingly pursued avenue [9]. One of the rate-limiting enzymes in the generation of extracellular adenosine is CD73, a GPI-anchored or soluble nucleotidase that catalyzes the dephosphorylation of AMP into adenosine. Whether CD73-generated adenosine is involved in regulation of B-1 innate B cell responses to *S*. *pneumoniae* infection is currently unknown.

On one hand, engaging the CD73-adenosine pathway might be able to suppress potentially harmful inflammatory host responses during pneumococcal bacterial pneumonia, as recently reported [13]. On the other hand, CD73-derived adenosine may be required for effective antibody responses following vaccination. Accordingly, in vitro studies revealed that CD73 expression promotes class switch recombination in B-2 B cells [14]. Furthermore, because CD73 expression in lymphocytes is developmentally regulated, CD73 levels on B cells are low in young infants and in patients with common variable immunodeficiency, two patient populations highly prone to *S*. *pneumoniae* infections [14, 15]. The objective of this study was thus to investigate the importance of CD73 expression for innate B cell responses to *S*. *pneumoniae* following vaccination.

Murine B-1 B cells have been previously reported to express CD73 [16, 17]. We tested the hypothesis that CD73 potentiates antibody effector responses of B-1 B cells and responsiveness to Pneumovax 23 vaccination. Using CD73-deficient and A2a-deficient knockout (KO) mice, we report that the CD73-A2a adenosine receptor axis enhances early IgM to IgG3 isotype switch responses in innate B cells in vivo, but is not critical for the control of *S*. *Pneumoniae* challenge following Pneumovax 23 vaccination in mice. Importantly, we also demonstrate that administration of an A2a adenosine receptor agonist in young mice is sufficient to enhance IgM to IgG3 isotype switch following Pneumovax 23 vaccination and improve the control of *S*. *pneumoniae* infection.

## Materials and methods

### Mice

C57BL/6J WT mice were obtained from the Jackson Laboratory. C57BL/6N WT mice were maintained at the CRCHUM. CD73 KO (C57BL/6J background) and A2a KO (C57BL/6N) mice were obtained from Dr. Linda F. Thompson (Oklahoma Medical Research Foundation) and from Dr. Jiang-Fan Chen (Boston University School of Medicine) respectively and were maintained at the CRCHUM. Animals were housed in groups of 5 per cage. All experiments were approved by the Animal Protection Committee of the CRCHUM.

### Antibodies and reagents

Pneumovax 23 (Merck) was obtained from CHUM pharmacy. PPS3 was obtained from ATCC. PC-BSA was obtained from Biosearch Technologies. Fluorochrome conjugated antibodies to rat anti-mouse CD19 (1D3), CD5 (53–7.3), CD23 (B3B4), CD73 (TY/11.8), CD43 (S7), CD21/35 (7G6), rat IgG1,κ isotype control unconjugated mouse CD16/32 (24G2), and rat IgG1,κ anti-mouse IgA (C10-3) were obtained from BD Biosciences. FITC-conjugated goat anti-rat IgG was obtained from eBioscience. CGS 21680 hydrochloride was obtained from Tocris Bioscience. *S*. *pneumoniae* strain WU-2 was a kind gift of Dr. David Briles (The University of Alabama at Birmingham). The bacteria were grown in Todd-Hewitt broth supplemented with 0.5% yeast extract and glycerol frozen stocks were kept at -80 °C.

### FACS

Single cell suspensions from spleen and peritoneal lavage (10^7^ cells/mL) were pre-incubated with mouse Fc Block (unconjugated CD16/32) for 15 minutes and then stained with fluorochrome conjugated antibodies for 30 minutes at 4°C. Cells were analyzed using a BD FACSDiva cytometer. Fluorescence minus one CD73 staining in CD73 KO mice or isotype control staining were used as negative controls.

### Immunizations and *S*. *pneumoniae* infection

8 to 12-week-old and 3-week-old mice were considered adult and young mice respectively. Adult mice were immunized intraperitoneally (i.p.) with Pneumovax 23 (2.9 μg in 100 μl of PBS). Young mice were immunized i.p. with 10 μg of Pneumovax 23 (in 100 μL of DPBS). Mice treated with the selective A2a agonist CGS 21680 received daily dose of 1mg/kg diluted in DPBS, injected i.p. for four weeks. Young mice were weighed weekly and the dose was adjusted consequently. Serum for determining Ig by ELISA was obtained prior to and at different time-points after immunization. For pneumococcal infections, non-vaccinated, young vaccinated and adult vaccinated mice were injected four weeks after immunization with 10, 5x10^3^ or 10^5^ CFU *S*. *pneumoniae* respectively i.p. and survival was monitored for one week. Animals were monitored for the development of the following clinical signs (dehydration, eye discharge, abnormally rapid breathing, hunched back, hypoactivity (grade 1: normal activity; grade 2: slight hypoactivity; grade 3 significant hypoactivity; grade 4: failure to move), lack of righting reflex, and response to stimuli). Monitoring was done every 12 hours for the first 24 hours, and subsequently every 6 hours or, if clinical signs or greater than 10% body weight loss were observed, every 3 hours. Body weight was monitored every 12 hours during the first day and subsequently every 6 hours. In case of dehydration, animals were given 0.1 ml of saline solution sub-cutaneously and wet pieces of food pellets were put inside the cage. Animals that showed markedly depressed ability to move (grade 3 and 4), with or without lack of righting reflex or lack of response to stimuli or markedly altered breathing were immediately sacrificed. No animal death prior to reaching these clinical signs was observed, and no animals were found dead. Experiments were performed in an NC2 animal facility and all monitoring and manipulation of animals was done by NC2 trained personnel.

### Enzyme-linked immunosorbent assays

ELISAs were performed according to a modified, previously published protocol [18]. For antigen-specific antibodies, nunc maxisorp plates were either coated overnight with pneumococcal polysaccharide type 3 (PPS3) (10 μg/ml), or Phosphorylcholine (PC)-BSA (5 μg/mL). For immunoglobulin levels, plates were coated overnight with goat anti-mouse IgG (10 μg/ml) or goat anti-mouse IgM (10 μg/ml). Blocking was done for 2 hours (200μL per well) at RT in PBS-0.1% Tween20-5% BSA. Samples were diluted in PBS-0.1% Tween20-0.1% BSA. For PPS3 ELISA, samples were preincubated for 60 minutes with CWPS (10μg/ml). Samples were plated overnight at 50 μL per well. HRP-conjugated anti-mouse IgM, IgG1, IgG2a, IgG2b, IgG3 and IgA detection antibodies (1:2000 dilution; Southern Biotech, Birmingham, AL) were added for 2 hours at room temperature (50 μL per well), followed by tetramethylbenzidine (TMB) substrate (50 μl per well). Reaction was stopped after 15 minutes with 2N hydrochloric acid (HCl) (50μl per well). Absorbance was read at 450 and corrected at 570 nm on a Versamax microplate reader. All steps were separated with washes (3X) made with PBS-0.1% Tween20. Samples were run in duplicates or triplicates. Analysis of ELISAs was done by determining OD (450–570). Each titer for a given mouse and ELISA was calculated as the inverse of the lowest dilution where OD readings are above 0.5 (at least 10-fold above background). Data from multiple experiments were analyzed by comparing the mean reciprocal titers of all mice included in WT, CD73 KO or A2a KO groups.

### Immunofluorescence for gut IgA

Terminal ileum tissue was harvested from 8-week-old WT and CD73 KO mice, frozen in OCT media, sectioned on Superfrost Plus slides (Fisher; 12-550-15) and stored at -80°C. Slides were thawed at room temperature for 30 minutes, fixed in 4% PFA, washed in PBS, blocked in DAKO protein blocker for 1 hour and stained with anti-mouse IgA primary antibody for 2 hours at room temperature. Sections were then washed in PBS and incubated with the FITC-conjugated goat anti-rat IgG secondary antibody for 2 hours at room temperature. Slides were mounted with Prolong Gold plus DAPI (Invitrogen) and images were taken using a Nikon microscope with a X20 objective.

### Statistical analysis

Data are shown as means +/- SEM. Statistics are performed by unpaired Student’s T test or one-way ANOVA with Bonferroni correction when multiple comparisons were made and survival curves were assessed by the log-rank test.

## Results

### CD73 defines specific subsets of naïve and isotype-switched innate type B cells

**Fig 1:**
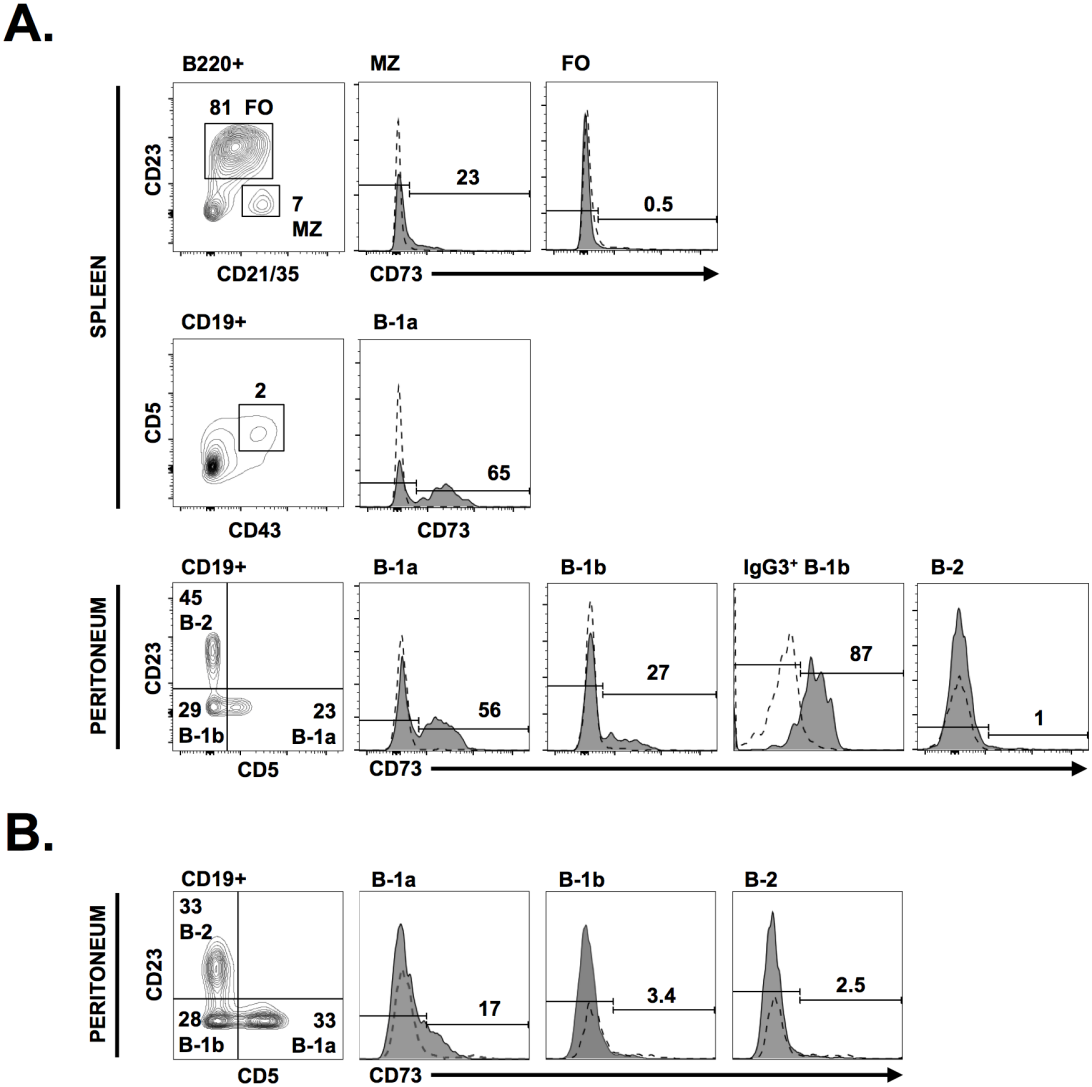
CD73 expression on B-1 B cells. (A) CD73 expression on distinct B cell subsets in adult 8 to 12-week-old mice (dashed lines show absence of staining in CD73 KO mice). (B) CD73 expression on B-1 B cells in young 3-week-old mice (dashed lines show isotype control). (FO: Follicular B cells, MZ: Marginal zone B cells).

We first evaluated CD73 expression in adult mouse B cells. In agreement with previous studies [16], we observed high expression of CD73 on a subset of peritoneal B-1a (CD19^+^ CD5^+^ CD23^-^) and B-1b cells (CD19^+^ CD5^-^ CD23^-^), splenic B-1a (CD19^+^ CD5^+^ CD43^+^) and marginal zone (MZ) (CD19^+^ CD21^hi^ CD23^low^) B cells (Fig 1A). Consistent with a role for CD73 in isotype class-switching [14], the vast majority (~87%) of isotype-switched (IgG3^+^) B-1b B cells expressed CD73 (Fig 1A). In young mice (3-week-old), however, the majority of B-1a and B-1b B cells were negative for CD73 (Fig 1B), consistent with prior reports of low CD73 expression on B cells from young infants [15].

### Normal B cell development and steady state antibody levels in CD73 KO mice

**Fig 2:**
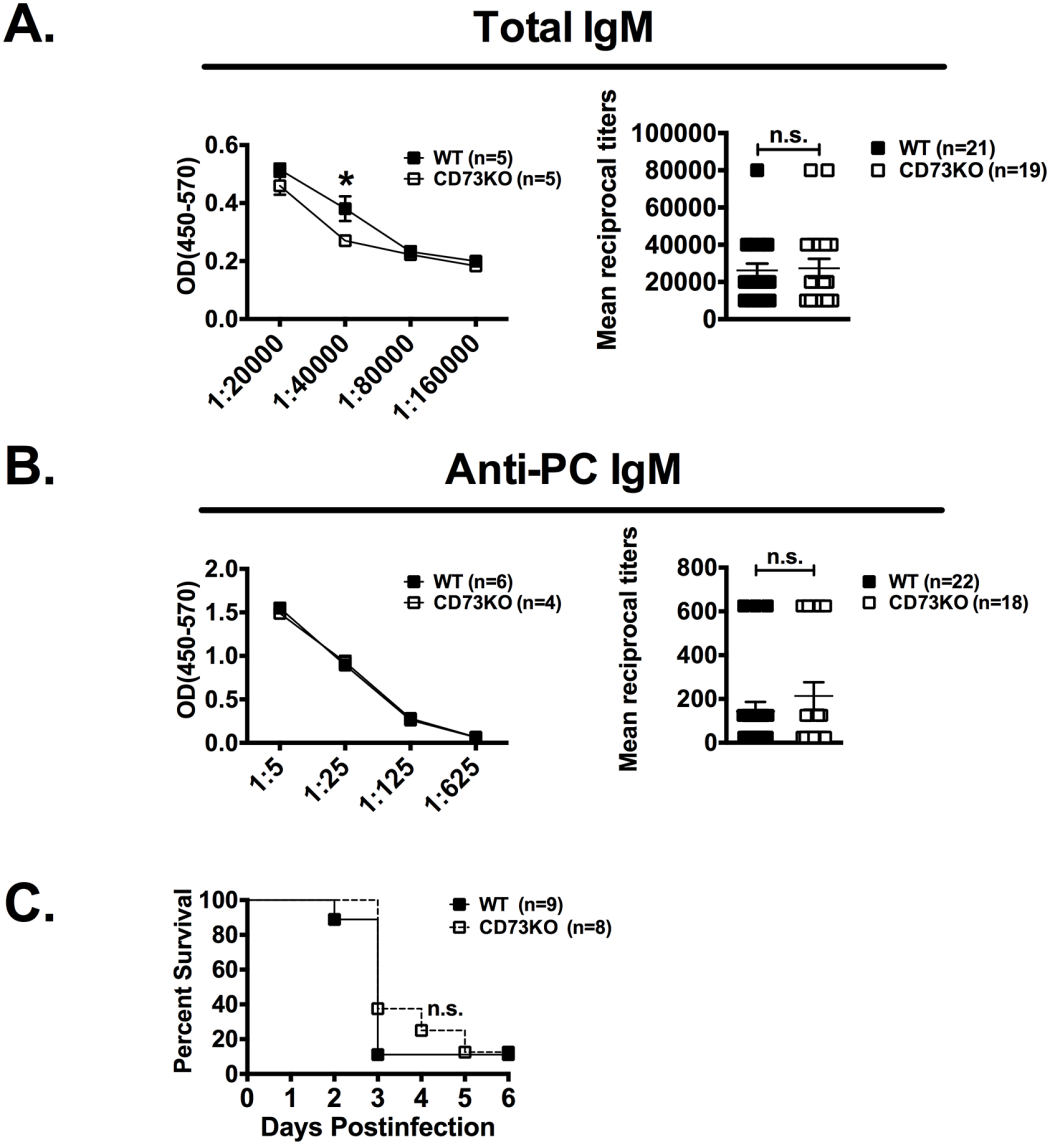
Preserved steady-state functions of B-1 B cells in CD73-deficient mice. (A) Total serum IgM levels were measured in adult WT and CD73 KO mice (8 to 12-week-old). Titration curves of one experiment are shown (means ± standard errors; *: p<0.05 by Student’s T test). Bar graphs were generated from three independent experiments (mean reciprocal titers ± standard errors are shown; n.s.: not significant; by Student T test). (B) Serum anti-phosphorylcholine (PC) IgM levels were measured in adult WT and CD73 KO mice (8 to 12-week-old). Titration curves of one experiment are shown. Bar graph were generated from three independent experiments (mean reciprocal titers ± standard errors are shown; n.s: not significant; by Student T test). (C) Naïve adult WT and CD73 KO mice (8 to 12-week-old) were injected i.p. with 10 CFU of *S*. *pneumoniae* and survival monitored over time (n.s: p>0.05 by log-rank test).

We next compared the steady-state levels of the various B cell subsets in adult WT and CD73-deficient (KO) mice. We observed no statistical difference in absolute numbers of B-1 and B-2 cells (Table 1), supporting that CD73 is not required for their development. We next investigated the role of CD73 in innate B-1 B cell responses. B-1 B cells are the source of the natural antibody pool (e.g. anti-PC Ab targeting *S*. *pneumoniae* phosphorylcholine) [19], and in this way, contribute to a major portion of serum IgM. This natural antibody pool controls to some extent *S*. *pneumoniae* bacteremia in unvaccinated mice [2]. We measured total serum IgM and anti-PC natural IgM levels in WT and CD73 KO mice. As shown in Fig 2A and 2B, no difference was observed between WT and CD73 KO mice. Because B-1 cells, especially the B-1b subset, have been shown to efficiently isotype switch to IgA-producing cells in vitro and in vivo [20, 21], we also compared gut-associated IgA levels, but observed no difference between WT and CD73 KO mice (Panel A in S1 Fig). Consistent with these results, no difference in survival was observed in WT versus CD73 KO mice following low-dose *S*. *pneumoniae* challenge (Fig 2C). Taken together, our data strongly suggest preserved steady-state functions of B-1 B cells in CD73-deficient mice.

**Table 1:** CD73 KO mice have similar numbers of major B cell subsets.

Tissue	Phenotype		Absolute Cell number (x10^6^)		Percentage of CD73^+^
			WT	CD73KO	WT
**Spleen**	**CD19**^**+**^		**37 ± 8**	**38 ± 9**	
	**FO**	**(B220^+^CD21^+^CD23^+^)**	**25 ± 2**	**27 ± 3**	**1.0 ± 0.4**
	**MZ**	**(B220^+^CD21^+^CD23^-^)**	**4.2 ± 0.2**	**4.7 ± 1.8**	**24.0 ± 0.3**
	**B-1a**	**(CD19^+^CD43^+^CD5^+^)**	**0.9 ± 0.2**	**0.9 ± 0.2**	**65 ± 4**
**Peritoneal Cavity**	**B-2**	**(CD19^+^CD23^+^CD5^-^)**	**0.60 ± 0.16**	**0.45 ± 0.17**	**6 ± 1**
	**B-1a**	**(CD19^+^CD23^-^CD5^+^)**	**0.35 ± 0.30**	**0.30 ± 0.10**	**53 ± 1**
	**B-1b**	**(CD19^+^CD23^-^CD5^-^)**	**0.16 ± 0.03**	**0.19 ± 0.07**	**24 ± 8**

Peritoneal cells and splenocytes from 8 to 12-week-old WT and CD73 KO mice were analyzed by Flow Cytometry for the presence of indicated B cell subsets. Cell numbers were obtained by obtaining absolute cell count as well as cell percentages. At least five mice of each genotype were analyzed. (FO: Follicular B cells, MZ: Marginal zone B cells).

### Delayed IgM to IgG3 isotype switching but comparable susceptibility to *S*. *pneumoniae* challenge in CD73 KO mice following Pneumovax 23 vaccination

**Fig 3:**
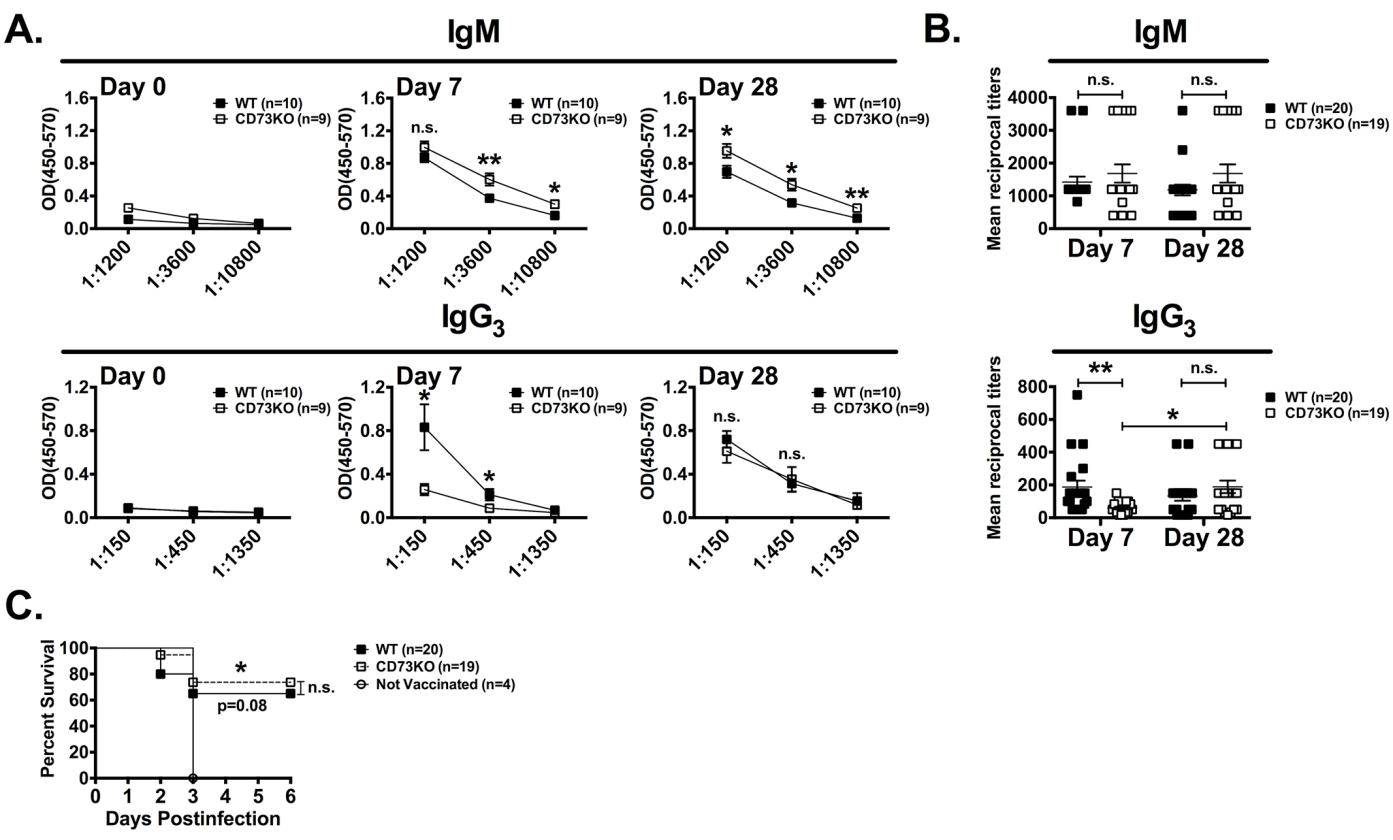
CD73-deficiency is associated with delayed IgG3 response after Pneumovax 23 vaccination. 8 to 12-week-old WT and CD73 KO mice were immunized i.p. with Pneumovax 23 and sera were analyzed by ELISA for anti-PPS3 specific Abs at day 0, 7 and 28 after vaccination. On day 28, mice were challenged by i.p. injection of *S*. *pneumoniae* (10^5^ CFU). (A) Anti-PPS3 IgM and IgG3 OD (450–570) readings are shown (means ± standard errors are shown; *: p<0.05; **: p<0.01 by Student’s T test). (B) Combined results from 2 experiments pooling 20 WT and 19 CD73 KO mice showing IgM and IgG3 mean reciprocal titers at day 7 and 28 (means ± standard errors are shown; **: p<0.01; by One-way ANOVA; brackets are shown when significantly different from day 7 and 28 for a given group). (C) Survival of infected mice was monitored (*: p<0.05 by log-rank test; survival was compared to non-vaccinated mice, unless indicated by brackets; n.s: not significant).

Induced T cell independent IgG (especially IgG3 in mice) and IgM produced by innate B cells play an important role in the immune control of *S*. *Pneumoniae* [2, 18]. We therefore compared IgG3 and IgM production following Pneumovax 23 vaccination in WT and CD73 KO mice. We also compared the ability of vaccinated WT and CD73 KO mice to control *S*. *pneumoniae* infection. Antibody responses were measured against PPS3, a major component of the *S*. *pneumoniae* strain WU2 capsule [22] that is included in the Pneumovax 23 vaccine. As shown in Fig 3, vaccination of adult CD73 KO mice with Pneumovax 23 induced a slightly higher PPS3-specific IgM response and a lower IgG3 response compared to WT mice in the first week following vaccination. The anti-PPS3 IgG3 response however normalized at later time-points (Fig 3A and 3B). These data thus indicated a delayed, yet effective, IgM to IgG3 isotype switching in CD73-deficient mice vaccinated with Pneumovax 23. To assess if this delay in isotype switching was associated to a decreased protective response, we challenged vaccinated mice with a lethal high-dose of S. *pneumoniae*. As shown in Fig 3C, survival rates in adult WT and CD73 KO mice were similar following challenge (Fig 3C). Our data thus demonstrated that CD73 was not required for protective innate B cell antibody responses against *S*. *pneumoniae*.

### IgG3 delay following Pneumovax 23 vaccination in A2aR KO mice

**Fig 4:**
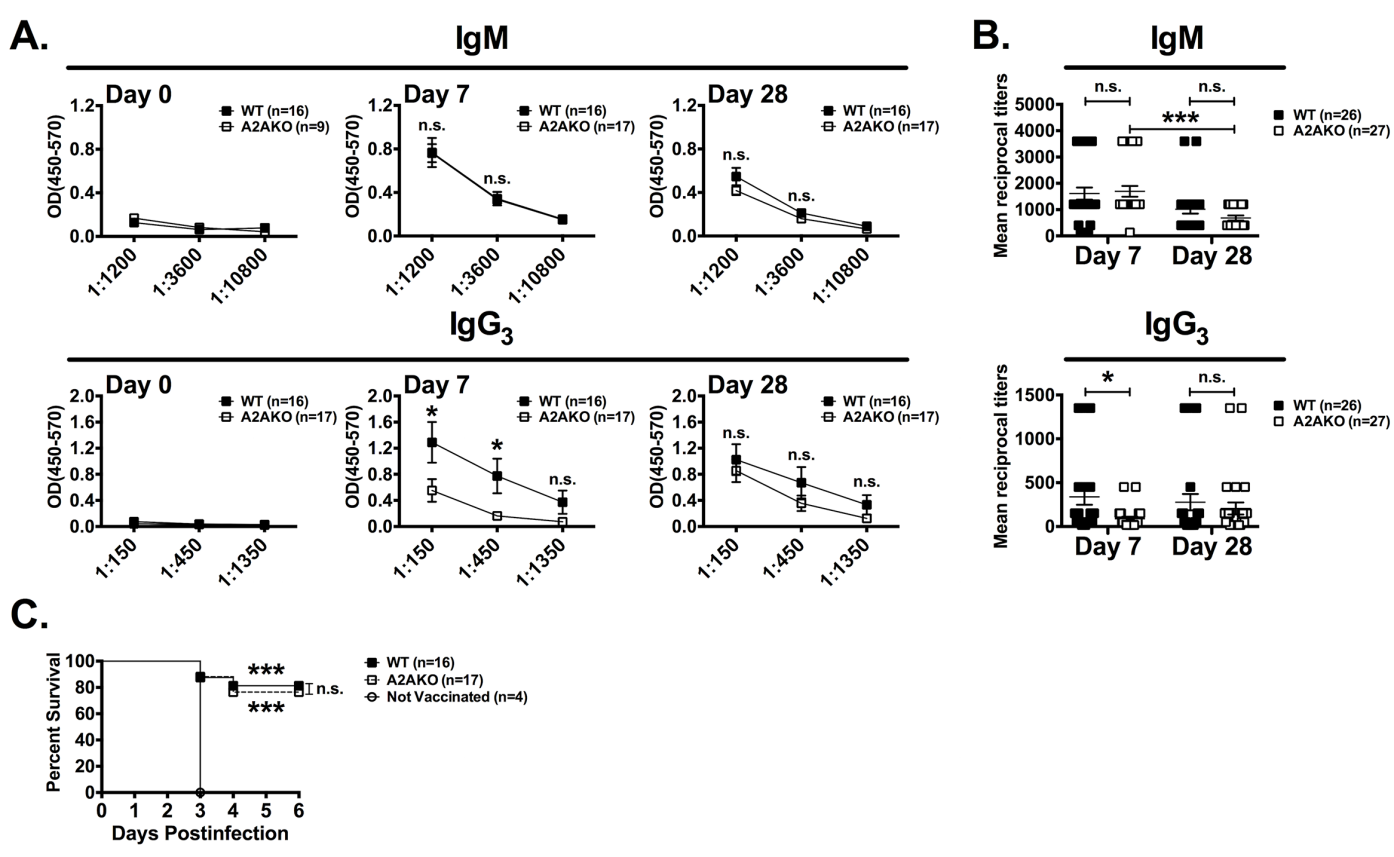
A2aR-deficiency is associated with delayed IgG3 response after Pneumovax 23 vaccination. 8 to 12-week-old WT and A2a KO mice were immunized i.p. with Pneumovax 23 and sera were analyzed by ELISA for anti-PPS3 specific Abs on days 0, 7 and 28 after vaccination. On day 28, mice were challenged by i.p. injection of *S*. *pneumoniae* (10^5^ CFU). (A) Anti-PPS3 IgM and IgG3 OD (450–570) readings are shown (means ± standard errors are shown; *: p<0.05; **: p<0.01 by Student’s T test). (B) Combined results from 2 experiments pooling 26 WT and 27 A2a KO mice showing IgM and IgG3 mean reciprocal titers at day 7 and 28 (means ± standard errors are shown; *: p<0.05; by One-way ANOVA; brackets are shown when significantly different from day 7 and 28 for a given group). (C) Survival of infected mice was monitored (*: p<0.05 by log-rank test; survival was compared to non-vaccinated mice, unless indicated by a bracket; n.s: not significant).

In order to test if the delay in Pneumovax induced IgG3 production in CD73 KO mice is a consequence of abrogated adenosine receptor engagement, we tested Pneumovax 23 antibody response in A2aR-deficient mice. The A2a receptor is ubiquitously expressed, and many biologic effects of adenosine, including the effect of adenosine on isotype switching during T cell dependent B cell responses [23, 24] are mediated via this receptor. A2aR-deficient mice show preserved frequencies and numbers of innate B-1 B cells (S2 Fig). Following Pneumovax 23 vaccination of A2aR KO mice we observed normal kinetics of PPS3 specific serum IgM but a delay of PPS3 specific IgG3 (Fig 4A and 4B). To rule out the possibility that the IgG3 response is simply skewed toward toward another IgG isotype, we measured PPS3 specific IgG1, IgG2a and IgG2b, but did not detect any increase of these isotypes. In addition PPS3 specific IgA levels were similarly increased in serum of vaccinated A2aR KO mice (S3 Fig). Similar to CD73 KO mice, PPS3 specific IgG3 serum levels were normal by 4 weeks, and A2aR-deficient mice were protected against a lethal live *S*. *pneumoniae* challenge (Fig 4C). These results suggest that the delay of IgG3 production in CD73 KO mice is a consequence of impaired A2a receptor engagement, and show that A2a receptor is also not required for Pneumovax 23 induced protective innate B cell responses.

### Administration of an A2a agonist enhances anti-PPS3 IgG3 Ab response in Pneumovax 23 vaccinated young mice and improves survival following *S*. *pneumoniae* challenge

**Fig 5:**
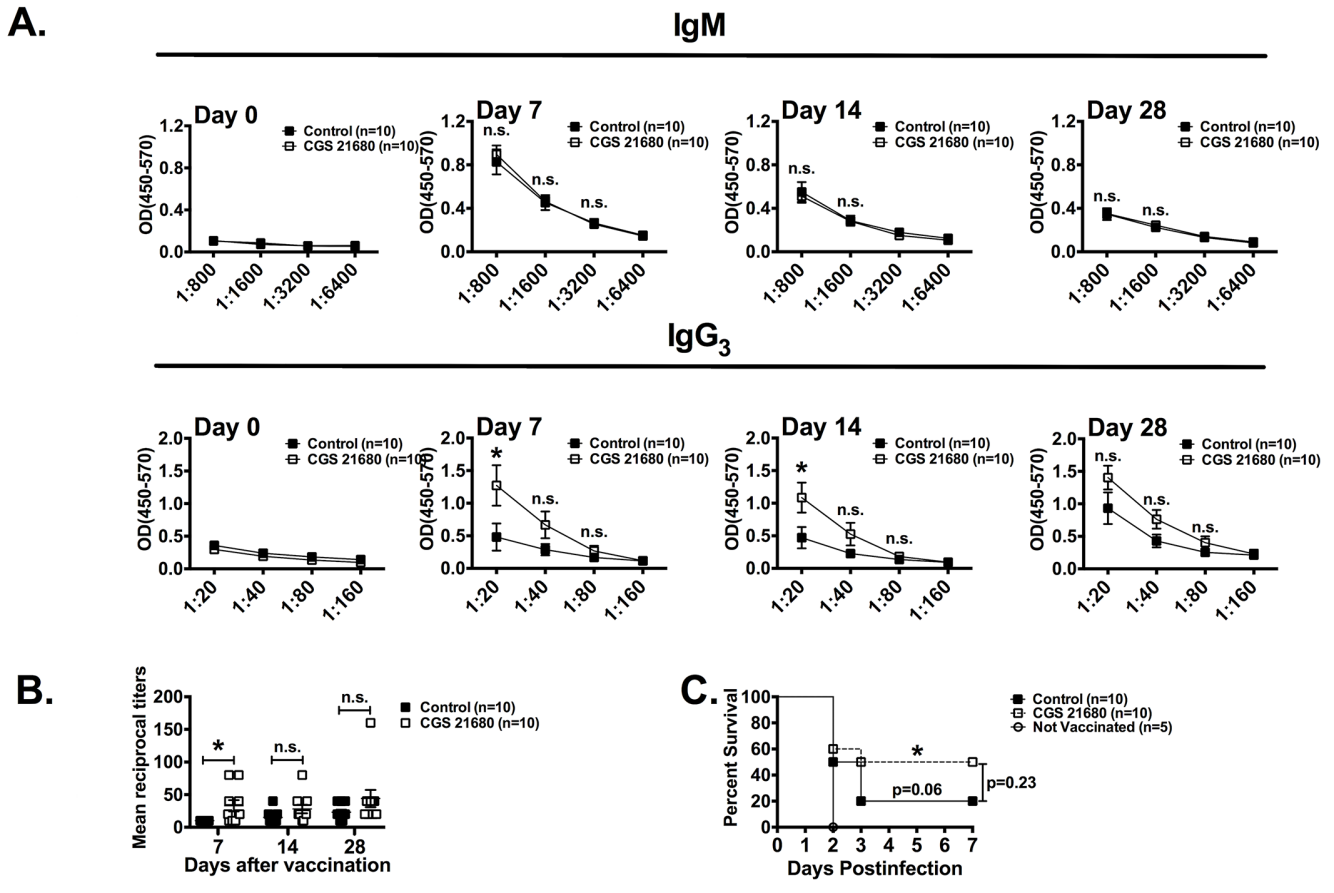
A2a receptor agonist enhances Pneumovax 23 responses in young mice. Young (3-week-old) WT mice were immunized i.p. with 10 μg of Pneumovax 23 and were subsequently treated daily with CGS 21680 or vehicle only DMSO (control) for 4 weeks as described in methods. Sera were analyzed by ELISA for anti-PPS3 specific Abs at day 0, 7, 14 and 28 after vaccination. On day 28, mice were challenged by i.p. injection of *S*. *pneumoniae* (5x10^3^ CFU). (A) Anti-PPS3 IgM and IgG3 OD (450–570) readings are shown (means ± standard errors are shown; *: p<0.05; **: p<0.01 by Student’s T test). (B) IgG3 mean reciprocal titers at day 7, 24 and 28 showing individual mice (means ± standard errors are shown; *: p<0.05; by One-way ANOVA). (C) Survival of infected mice (*: p≤0.05 by log-rank test; n.s.: p>0.05; survival was compared to non-vaccinated mice unless indicated by a bracket).

Children under 2 years of age and young mice respond poorly to Pneumovax 23 vaccination [25]. Based on our observation that CD73 is poorly expressed on B-1 B cells in young mice and that CD73 and the A2aR promote isotype switching, we hypothesized that pharmacological agonists of the A2aR receptors may enhance the protective effects of Pneumovax 23 in this population. We thus investigated whether administration of an A2a adenosine receptor agonist (CGS 21680) could enhance antibody responses following Pneumovax 23 vaccination in young mice. As shown in Fig 5A and 5B, treatment with the A2a agonist significantly increased mean IgG3 levels at day 7 and 14 post-vaccination (Fig 5A and 5B). Notably, this increase in IgG3 titers, although still lower than in vaccinated adult mice (Figs 5B and 3B and 4B) reflected a higher percentage of mice that responded to the vaccine (i.e. 6/10 mice in the CGS-treated group compared to 3/10 in the control group on day 28; Fig 5B). We next challenged the vaccinated mice with a lethal high-dose of *S*. *pneumoniae*. Consistent with prior studies [23, 25], vaccination of young WT mice with Pneumovax 23 failed to significantly protect them from *S*. *pneumoniae* infection. In contrast, co-administration of Pneumovax 23 and the A2a receptor agonist significantly increased survival of young mice compared to non-vaccinated mice (Fig 5C). Our study thus demonstrated that pharmacological activation of A2a adenosine receptor signaling is an effective means to promote class-switch recombination and enhance the protective effects of pneumococcal polysaccharide vaccination in young mice.

## Discussion

We here addressed the role of the adenosine-generating ecto-enzyme CD73 on innate B cell responses during streptococcal infections. Consistent with prior in vitro work that revealed a role for CD73 in class-switch recombination [14], we demonstrated that CD73 promoted IgM to IgG3 isotype switching in mice vaccinated with Pneumovax 23. However, while CD73 expression accelerated IgG3 responses, CD73-deficient mice eventually mounted similar levels of antigen-specific IgG3 as WT mice. Hence, CD73 is not required for in vivo isotype switching, at least in response to Pneumovax 23. Our data further support correlative observations previously made in humans, where functionally compromised neonatal B cells were shown to lack CD73 expression [15] and adult isotype-switched B cells were shown to be enriched for CD73^+^ cells [14].

Our observations using A2a-deficient KO mice suggest that the contribution of CD73 toward IgG3 production occurred through its enzymatic activity and through the engagement of the A2a receptor. Our data also indicate that delayed IgG3 production is not a consequence of skewing of IgG3 Ab response towards other IgG isotypes and that Pneumovax induced IgA production is not compromised.

The biologic basis of the isotype switch delay observed in CD73-deficient mice warrants further investigation. IgM to IgG3 class switch recombination is induced by synergistic BCR and TLR signalling [26], but is also modulated by cytokines, such as IL-21 [27], at least in human B cells. Interestingly, IL-21 is known to upregulate CD73 on B and T cells [14]. Adenosine generated by CD73 has been shown to modulate cytokine production in macrophages [28]. It is possible that altered cytokine production lies at the heart of the isotype switch delay in CD73-deficient mice. Alternatively, since CD73 is known to be involved in the regulation of lymphocyte homing [29], the lack of extracellular CD73-generated adenosine could also impact the homing of B-1 B cells to the spleen, which is a component of an efficient antibody response [3]. Proper homing of B cells to the marginal zone is regulated by the chemokines and chemokine receptors. Interestingly, adenosine has been proposed to modulate the CXCL12/CXCR4 axis [30] which may be relevant since B cell deficiency of CXCR4 leads to a decreased size of the marginal zone [29] as well as decreased TI antibody responses [31]. Alternatively, the CD73/A2a axis could regulate the rate of expansion of isotype switched B cells. Preliminary experiments assessing in vitro B-1 B cell IgG3 Ab production failed to show a difference between WT and CD73KO mice, suggesting an abnormal in-vivo niche for IgG3 producing cells in CD73KO mice.

The observation that we could increase Pneumovax 23-induced IgG3 levels and improve protection against *S*. *pneumoniae* challenge by administering an A2a receptor agonist, suggests that modulation of the extracellular adenosine pathway is a potential avenue for increasing responsiveness to Pneumovax 23 in vulnerable populations, such as young infants and elderly. B cells in very young individuals show several deficiencies and are functionally compromised. B cells from newborns have very low levels of CD73 and low levels of the transmembrane activator and calcium modulator and cyclophilin ligand interactor (TACI; receptor for BAFF and APRIL), which plays a key role in efficient antibody responses against polysaccharide vaccines [32]. Interestingly, recent studies demonstrated that A2a adenosine receptor negatively regulated isotype switched IgG1 germinal center (GC) responses [23], likely via the suppression of T follicular helper cell differentiation [23, 24]. The impact of A2a receptor agonist treatment on antibody responses may thus vary depending on the cells that are targeted and mechanisms of B cell activation.

The introduction of pneumococcal conjugate vaccines (PCVs) has significantly reduced the incidence of invasive pneumococcal disease caused by vaccine included serotypes [33], particularly in children. However, strains not included in the PCV vaccines still cause considerable disease, especially in susceptible populations. Therefore, administration of Pneumovax 23, which has a wider serotype coverage, is recommended in children and adults with conditions that put them at an increased risk of disease and in adults 65 years or older. For these individuals, optimization of anti-Pneumovax 23 vaccine response still remains a priority. The effect of CD73/A2a pathway modulation in the context of PCV however needs to be carefully assessed, as A2a agonist rather than antagonist has been described to enhance T cell dependent germinal center reactions (21). Timing of PCV and Pneumovax 23 administration and of any adenosinergic pathway modulation may be critical. In conclusion, our study clarified the role of CD73 and the A2a adenosine receptor on innate B cell isotype-switching and identified A2a adenosine receptor as a potential target to enhance the protective activity of Pneumovax 23 against *S*. *pneumoniae* infections. Therapeutic targeting of A2a receptor could be attempted in combination with the targeting of additional pathways already known to modulate anti-Pneumovax 23 responses, such as PD-1 [22], TLR [34], or IL-7/IL7-R [25].

## Supporting information

S1 Fig CD73 KO mice have normal Ig levels. 8 to 12-week-old WT and CD73 KO mice were assessed for Ig levels. (A) Detection of IgA-secreting cells in the gut by immunohistochemistry. Area of anti-IgA FITC staining was normalized to DAPI. Data were generated from 40 fields from WT and 20 fields from CD73 KO mice. (B) Serum IgG and IgG3 levels were determined by ELISA. (n.s.: p>0.05, unpaired Student’s T test; means ± standard errors are shown).
(TIFF)

S2 Fig A2a-deficient KO mice have preserved Peritoneal B-1 B cell populations. Peritoneal cells were pooled from 14 WT and 12 A2a KO mice, and were analyzed by FACS for B-1 B cell populations. Numbers represent percentages and cell numbers (in parentheses, expressed as cells per mouse).
(TIFF)

S3 Fig A2a KO vaccinated mice have normal PPS3 specific IgA levels. 16 and 17, 8 to 12-week-old, WT and A2a KO mice respectively were assessed for PPS3 specific IgA, IgG1, IgG2a and IgG2b levels 1 week after Pneumovax immunization. Serum levels were determined by ELISA. (n.s.: p>0.05, unpaired Student’s T test; means ± standard errors are shown).
(TIFF)

## References

[1] Ash SY, Sheffield JV. Pneumococcus. Med Clin North Am. 2013;97(4):647–666, x–xi. doi: 10.1016/j.mcna.2013.03.005 23809718 10.1016/j.mcna.2013.03.005

[2] Haas KM, Poe JC, Steeber DA, Tedder TF. B-1a and B-1b cells exhibit distinct developmental requirements and have unique functional roles in innate and adaptive immunity to S. pneumoniae. Immunity. 2005;23(1):7–18. doi: 10.1016/j.immuni.2005.04.011 16039575 10.1016/j.immuni.2005.04.011

[3] Martin F, Oliver AM, Kearney JF. Marginal zone and B1 B cells unite in the early response against T-independent blood-borne particulate antigens. Immunity. 2001;14(5):617–629. 11371363 10.1016/s1074-7613(01)00129-7

[4] Leggat DJ, Khaskhely NM, Iyer AS, Mosakowski J, Thompson RS, Weinandy JD, et al Pneumococcal polysaccharide vaccination induces polysaccharide-specific B cells in adult peripheral blood expressing CD19(+)CD20(+)CD3(-)CD70(-)CD27(+)IgM(+)CD43(+)CD5(+)/(-). Vaccine. 2013;31(41):4632–4640. doi: 10.1016/j.vaccine.2013.07.030 23911852 10.1016/j.vaccine.2013.07.030 PMC3810315

[5] Roth A, Glaesener S, Schutz K, Meyer-Bahlburg A. Reduced Number of Transitional and Naive B Cells in Addition to Decreased BAFF Levels in Response to the T Cell Independent Immunogen Pneumovax(R)23. PLoS One. 2016;11(3):e0152215 doi: 10.1371/journal.pone.0152215 27031098 10.1371/journal.pone.0152215 PMC4816312

[6] Rothstein TL, Quach TD. The human counterpart of mouse B-1 cells. Ann N Y Acad Sci. 2015;1362:143–152. doi: 10.1111/nyas.12790 25988790 10.1111/nyas.12790

[7] Rothstein TL. Natural Antibodies as Rheostats for Susceptibility to Chronic Diseases in the Aged. Front Immunol. 2016;7:127 doi: 10.3389/fimmu.2016.00127 27092140 10.3389/fimmu.2016.00127 PMC4823301

[8] Daniels CC, Rogers PD, Shelton CM. A Review of Pneumococcal Vaccines: Current Polysaccharide Vaccine Recommendations and Future Protein Antigens. J Pediatr Pharmacol Ther. 2016;21(1):27–35. doi: 10.5863/1551-6776-21.1.27 26997927 10.5863/1551-6776-21.1.27 PMC4778694

[9] Allard B, Beavis PA, Darcy PK, Stagg J. Immunosuppressive activities of adenosine in cancer. Curr Opin Pharmacol. 2016;29:7–16. doi: 10.1016/j.coph.2016.04.001 27209048 10.1016/j.coph.2016.04.001

[10] Antonioli L, Pacher P, Vizi ES, Hasko G. CD39 and CD73 in immunity and inflammation. Trends Mol Med. 2013;19(6):355–367. doi: 10.1016/j.molmed.2013.03.005 23601906 10.1016/j.molmed.2013.03.005 PMC3674206

[11] Allard B, Longhi MS, Robson SC, Stagg J. The ectonucleotidases CD39 and CD73: Novel checkpoint inhibitor targets. Immunol Rev. 2017;276(1):121–144. doi: 10.1111/imr.12528 28258700 10.1111/imr.12528 PMC5338647

[12] Vijayan D, Young A, Teng MWL, Smyth MJ. Targeting immunosuppressive adenosine in cancer. Nat Rev Cancer. 2017;17(12):709–724. doi: 10.1038/nrc.2017.86 29059149 10.1038/nrc.2017.86

[13] Bou Ghanem EN, Clark S, Roggensack SE, McIver SR, Alcaide P, Haydon PG, et al Extracellular Adenosine Protects against Streptococcus pneumoniae Lung Infection by Regulating Pulmonary Neutrophil Recruitment. PLoS Pathog. 2015;11(8):e1005126 doi: 10.1371/journal.ppat.1005126 26313746 10.1371/journal.ppat.1005126 PMC4552087

[14] Schena F, Volpi S, Faliti CE, Penco F, Santi S, Proietti M, et al Dependence of immunoglobulin class switch recombination in B cells on vesicular release of ATP and CD73 ectonucleotidase activity. Cell Rep. 2013;3(6):1824–1831. doi: 10.1016/j.celrep.2013.05.022 23770243 10.1016/j.celrep.2013.05.022

[15] Pettengill MA, Levy O. Circulating Human Neonatal Naive B Cells are Deficient in CD73 Impairing Purine Salvage. Front Immunol. 2016;7:121 doi: 10.3389/fimmu.2016.00121 27066009 10.3389/fimmu.2016.00121 PMC4812068

[16] Kaku H, Cheng KF, Al-Abed Y, Rothstein TL. A novel mechanism of B cell-mediated immune suppression through CD73 expression and adenosine production. J Immunol. 2014;193(12):5904–5913. doi: 10.4049/jimmunol.1400336 25392527 10.4049/jimmunol.1400336 PMC4321875

[17] Almishri W, Deans J, Swain MG. Rapid activation and hepatic recruitment of innate-like regulatory B cells after invariant NKT cell stimulation in mice. J Hepatol. 2015;63(4):943–951. doi: 10.1016/j.jhep.2015.06.007 26095178 10.1016/j.jhep.2015.06.007

[18] Haas KM, Blevins MW, High KP, Pang B, Swords WE, Yammani RD. Aging promotes B-1b cell responses to native, but not protein-conjugated, pneumococcal polysaccharides: implications for vaccine protection in older adults. J Infect Dis. 2014;209(1):87–97. doi: 10.1093/infdis/jit442 23964109 10.1093/infdis/jit442 PMC3864388

[19] Savage HP, Baumgarth N. Characteristics of natural antibody-secreting cells. Ann N Y Acad Sci. 2015;1362:132–142. doi: 10.1111/nyas.12799 26104151 10.1111/nyas.12799 PMC4679694

[20] Roy B, Shukla S, Lyszkiewicz M, Krey M, Viegas N, Duber S, et al Somatic hypermutation in peritoneal B1b cells. Mol Immunol. 2009;46(8–9):1613–1619. doi: 10.1016/j.molimm.2009.02.026 19327839 10.1016/j.molimm.2009.02.026

[21] Roy B, Brennecke AM, Agarwal S, Krey M, Duber S, Weiss S. An intrinsic propensity of murine peritoneal B1b cells to switch to IgA in presence of TGF-beta and retinoic acid. PLoS One. 2013;8(12):e82121 doi: 10.1371/journal.pone.0082121 24324757 10.1371/journal.pone.0082121 PMC3855760

[22] McKay JT, Egan RP, Yammani RD, Chen L, Shin T, Yagita H, et al PD-1 suppresses protective immunity to Streptococcus pneumoniae through a B cell-intrinsic mechanism. J Immunol. 2015;194(5):2289–2299. doi: 10.4049/jimmunol.1401673 25624454 10.4049/jimmunol.1401673 PMC4339454

[23] Abbott RK, Silva M, Labuda J, Thayer M, Cain DW, Philbrook P, et al The GS Protein-coupled A2a Adenosine Receptor Controls T Cell Help in the Germinal Center. J Biol Chem. 2017;292(4):1211–1217. doi: 10.1074/jbc.C116.764043 27974461 10.1074/jbc.C116.764043 PMC5270467

[24] Schmiel SE, Yang JA, Jenkins MK, Mueller DL. Cutting Edge: Adenosine A2a Receptor Signals Inhibit Germinal Center T Follicular Helper Cell Differentiation during the Primary Response to Vaccination. J Immunol. 2017;198(2):623–628. doi: 10.4049/jimmunol.1601686 27986907 10.4049/jimmunol.1601686 PMC5225048

[25] Shriner AK, Liu H, Sun G, Guimond M, Alugupalli KR. IL-7-dependent B lymphocytes are essential for the anti-polysaccharide response and protective immunity to Streptococcus pneumoniae. J Immunol. 2010;185(1):525–531. doi: 10.4049/jimmunol.0902841 20505146 10.4049/jimmunol.0902841

[26] Pone EJ, Zhang J, Mai T, White CA, Li G, Sakakura JK, et al BCR-signalling synergizes with TLR-signalling for induction of AID and immunoglobulin class-switching through the non-canonical NF-kappaB pathway. Nat Commun. 2012;3:767 doi: 10.1038/ncomms1769 22473011 10.1038/ncomms1769 PMC3337981

[27] Pene J, Gauchat JF, Lecart S, Drouet E, Guglielmi P, Boulay V, et al Cutting edge: IL-21 is a switch factor for the production of IgG1 and IgG3 by human B cells. J Immunol. 2004;172(9):5154–5157. 15100251 10.4049/jimmunol.172.9.5154

[28] Hamidzadeh K, Mosser DM. Purinergic Signaling to Terminate TLR Responses in Macrophages. Front Immunol. 2016;7:74 doi: 10.3389/fimmu.2016.00074 26973651 10.3389/fimmu.2016.00074 PMC4773587

[29] Salmi M, Jalkanen S. Homing-associated molecules CD73 and VAP-1 as targets to prevent harmful inflammations and cancer spread. FEBS Lett. 2011;585(11):1543–1550. doi: 10.1016/j.febslet.2011.04.033 21515268 10.1016/j.febslet.2011.04.033

[30] Serra S, Horenstein AL, Vaisitti T, Brusa D, Rossi D, Laurenti L, et al CD73-generated extracellular adenosine in chronic lymphocytic leukemia creates local conditions counteracting drug-induced cell death. Blood. 2011;118(23):6141–6152. doi: 10.1182/blood-2011-08-374728 21998208 10.1182/blood-2011-08-374728 PMC3342854

[31] Nie Y, Waite J, Brewer F, Sunshine MJ, Littman DR, Zou YR. The role of CXCR4 in maintaining peripheral B cell compartments and humoral immunity. J Exp Med. 2004;200(9):1145–1156. doi: 10.1084/jem.20041185 15520246 10.1084/jem.20041185 PMC2211858

[32] Kanswal S, Katsenelson N, Selvapandiyan A, Bram RJ, Akkoyunlu M. Deficient TACI expression on B lymphocytes of newborn mice leads to defective Ig secretion in response to BAFF or APRIL. J Immunol. 2008;181(2):976–990. 18606649 10.4049/jimmunol.181.2.976

[33] Balsells E, Guillot L, Nair H, Kyaw MH. Serotype distribution of Streptococcus pneumoniae causing invasive disease in children in the post-PCV era: A systematic review and meta-analysis. PLoS One. 2017;12(5):e0177113 doi: 10.1371/journal.pone.0177113 28486544 10.1371/journal.pone.0177113 PMC5423631

[34] Taillardet M, Haffar G, Mondiere P, Asensio MJ, Pleau-Pison T, Burdin N, et al Toll-like receptor agonists allow generation of long-lasting antipneumococcal humoral immunity in response to a plain polysaccharidic vaccine. J Infect Dis. 2010;202(3):470–479. doi: 10.1086/653739 20575660 10.1086/653739

