# Supplementary figures and images for: CD73-A2a adenosine receptor axis promotes innate B cell antibody responses to pneumococcal polysaccharide vaccination
Source: PLoS One. 2018 Jan 29;13(1):e0191973. doi: 10.1371/journal.pone.0191973 (PMC5788373; doi:10.1371/journal.pone.0191973)

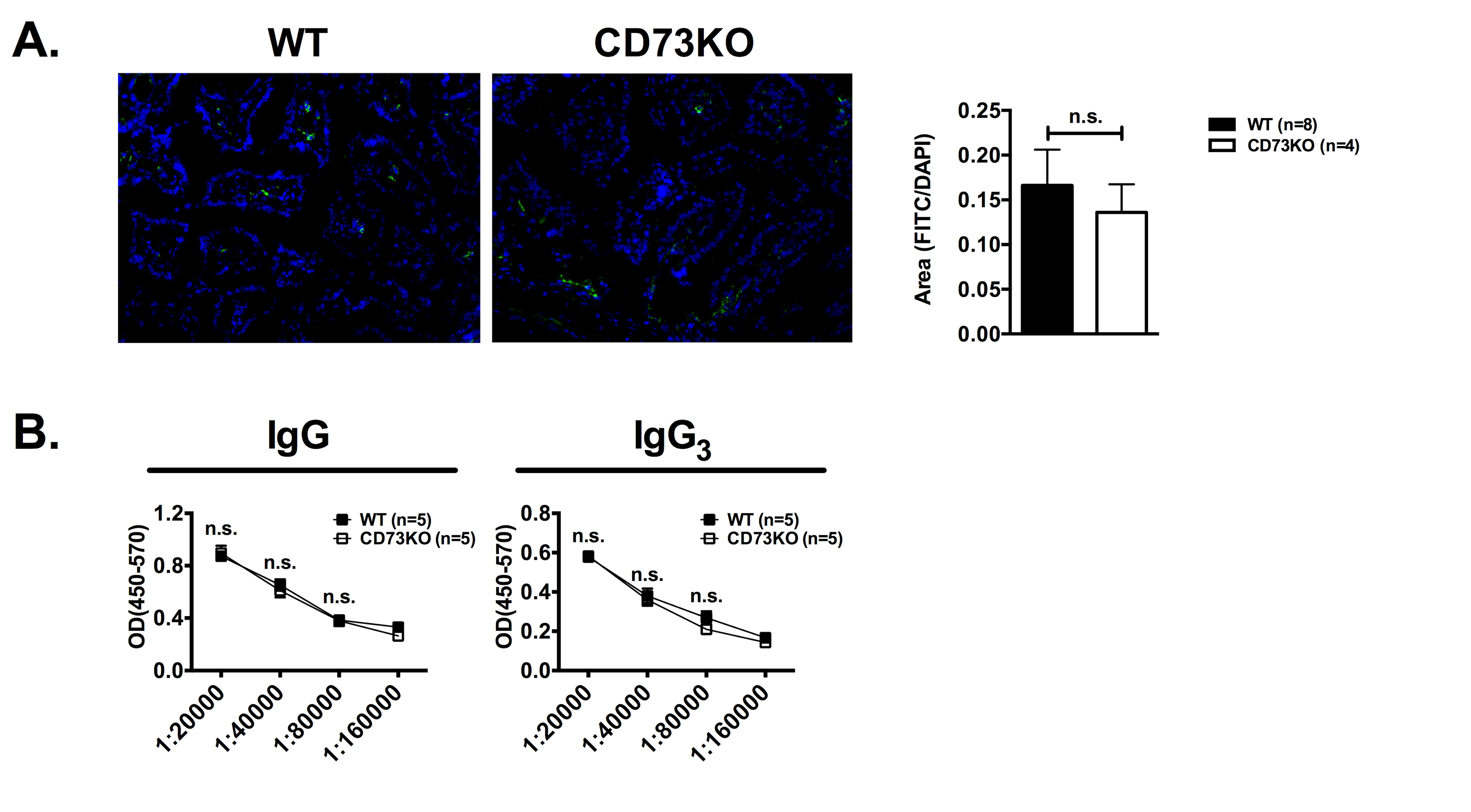

Supplement: S1 Fig — 8 to 12-week-old WT and CD73 KO mice were assessed for Ig levels. (A) Detection of IgA-secreting cells in the gut by immunohistochemistry. Area of anti-IgA FITC staining was normalized to DAPI. Data were generated from 40 fields from WT and 20 fields from CD73 KO mice. (B) Serum IgG and IgG3 levels were determined by ELISA. (n.s.: p>0.05, unpaired Student’s T test; means ± standard errors are shown). (TIFF) [file pone.0191973.s001.tiff]

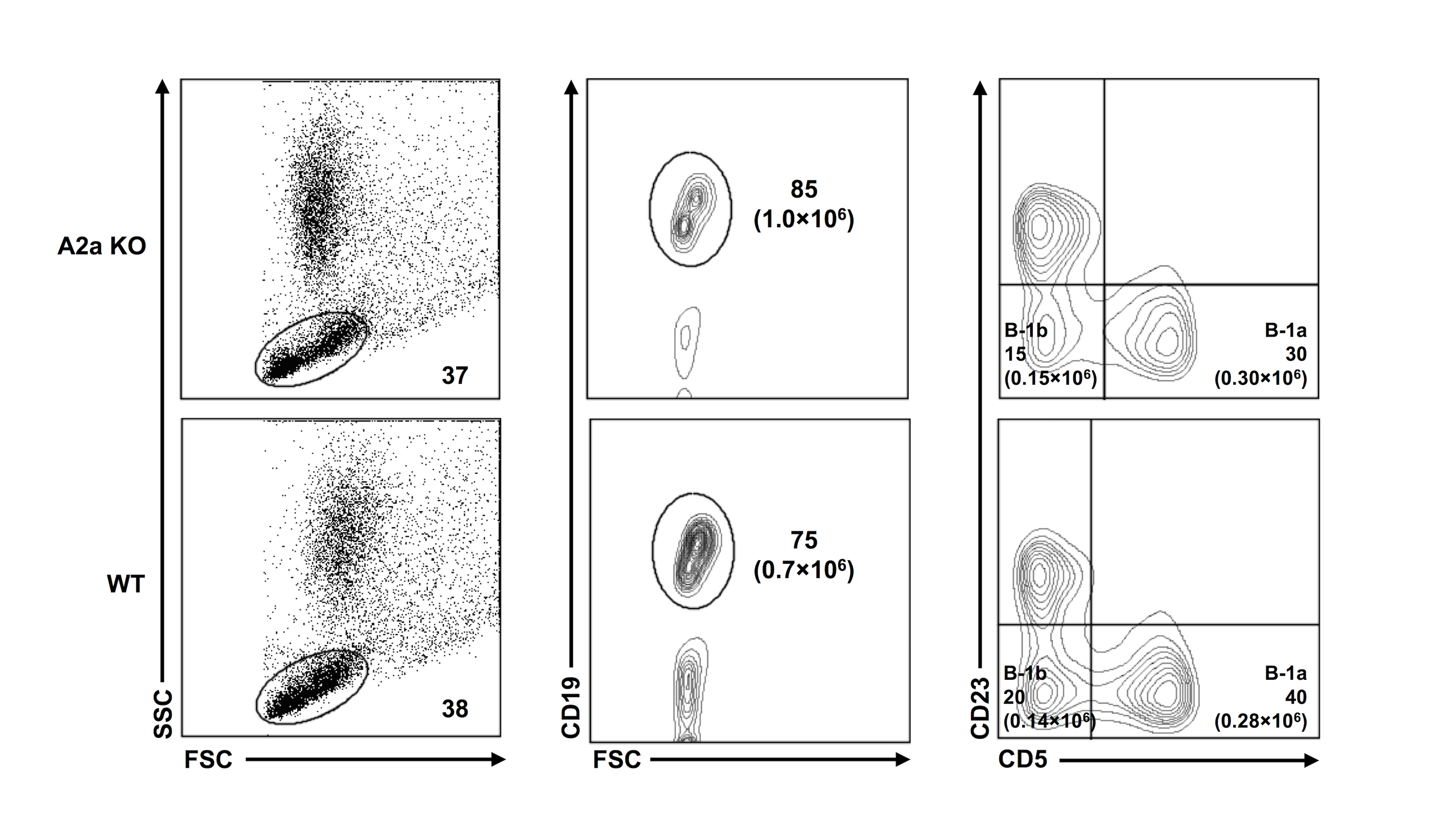

Supplement: S2 Fig — Peritoneal cells were pooled from 14 WT and 12 A2a KO mice, and were analyzed by FACS for B-1 B cell populations. Numbers represent percentages and cell numbers (in parentheses, expressed as cells per mouse). (TIFF) [file pone.0191973.s002.tiff]

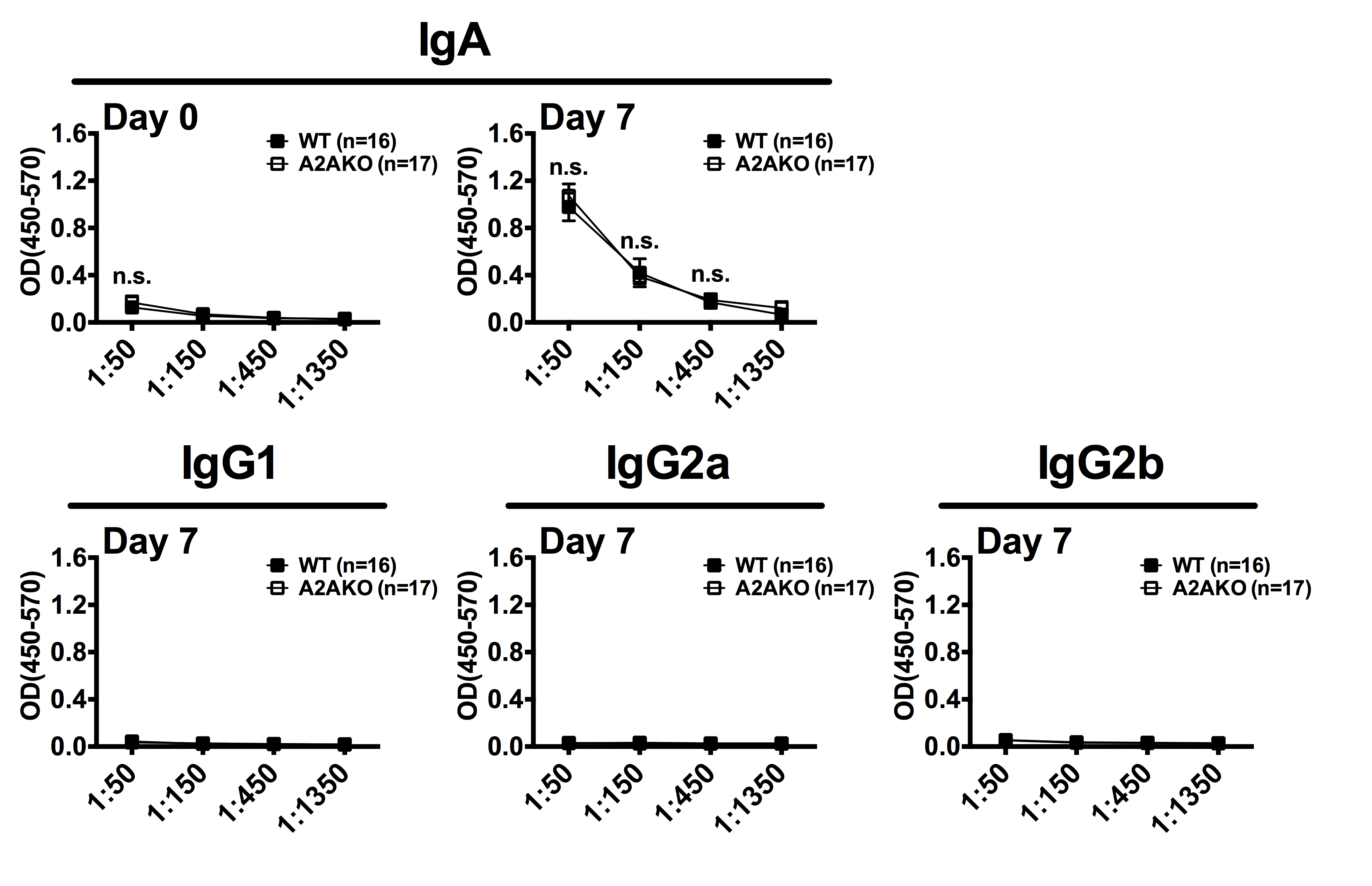

Supplement: S3 Fig — 16 and 17, 8 to 12-week-old, WT and A2a KO mice respectively were assessed for PPS3 specific IgA, IgG1, IgG2a and IgG2b levels 1 week after Pneumovax immunization. Serum levels were determined by ELISA. (n.s.: p>0.05, unpaired Student’s T test; means ± standard errors are shown). (TIFF) [file pone.0191973.s003.tiff]
